# AhR–ROR-γt complex is a therapeutic target for MAP4K3/GLK^high^IL-17A^high^ subpopulation of systemic lupus erythematosus

**DOI:** 10.1096/fj.201900105RR

**Published:** 2019-08-01

**Authors:** Huai-Chia Chuang, Yi-Ming Chen, Ming-Han Chen, Wei-Ting Hung, Huang-Yu Yang, Yang-Hao Tseng, Tse-Hua Tan

**Affiliations:** * Immunology Research Center, National Health Research Institutes, Zhunan, Taiwan;; † Division of Allergy, Immunology, and Rheumatology, Taichung Veterans General Hospital, Taichung, Taiwan;; ‡ Division of Allergy, Immunology, and Rheumatology, Taipei Veterans General Hospital, Taipei, Taiwan;; § Department of Medicine, Chang Gung Memorial Hospital, Taoyuan, Taiwan;; ¶ Department of Pathology and Immunology, Baylor College of Medicine, Houston, Texas, USA

**Keywords:** SLE, verteporfin, autoimmune disease

## Abstract

The cytokine IL-17A plays critical roles in the pathogenesis of autoimmune diseases. The frequencies of MAP kinase kinase kinase kinase 3 [also named germinal center kinase–like kinase (GLK)]-overexpressing T cells are correlated with disease severity of systemic lupus erythematosus (SLE). T-cell–specific GLK-transgenic mice develop spontaneous autoimmune responses through IL-17A. GLK signaling selectively stimulates IL-17A production in murine T cells through inducing aryl hydrocarbon receptor (AhR)–retinoic acid receptor–related orphan nuclear receptor-γt (ROR-γt) complex formation. Here, we investigated whether GLK-induced AhR–ROR-γt complex in T cells is a therapeutic target for human SLE. The population of GLK^+^IL-17A^+^ T cells was enhanced in the peripheral blood from patients with SLE compared with that of healthy controls using flow cytometry. The receiver operating characteristic curve analysis showed that increased GLK^+^IL-17A^+^ T-cell population in peripheral blood reflected an active stage of SLE. In addition, peripheral blood T cells from patients with SLE displayed induction of ROR-γt phosphorylation and the AhR–ROR-γt (and AhR–phosphorylated ROR-γt) complex. Moreover, we identified a small-molecule inhibitor, verteporfin, that inhibited GLK kinase activity and AhR–ROR-γt interaction. The small-molecule inhibitor verteporfin suppressed the disease severity in autoimmune mouse models and IL-17A production in T cells from patients with SLE. Collectively, the GLK-induced AhR–ROR-γt (and AhR–phosphorylated ROR-γt) complex is a therapeutic target for the GLK^high^IL-17A^high^ subpopulation of human patients with SLE.—Chuang, H.-C., Chen, Y.-M., Chen, M.-H., Hung, W.-T., Yang, H.-Y., Tseng, Y.-H., Tan, T.-H. AhR–ROR-γt complex is a therapeutic target for MAP4K3/GLK^high^IL-17A^high^ subpopulation of systemic lupus erythematosus.

The proinflammatory cytokine IL-17A plays critical roles in the pathogenesis of autoimmune diseases, such as psoriasis, rheumatoid arthritis (RA), psoriatic arthritis, and ankylosing spondylitis. Moreover, serum IL-17A levels and peripheral IL-17A–producing T cells are increased in systemic lupus erythematosus (SLE) (1–5). Induction of IL-17A occurs in the renal biopsies from patients with SLE (6). To date, IL-17A blockade shows therapeutic effects on several autoimmune diseases, including psoriasis, RA, psoriatic arthritis, and ankylosing spondylitis (7, 8); however, development of therapeutic treatment for SLE is very challenging because of disease complexity and heterogeneity (1). Thus, understanding the mechanisms controlling IL-17A induction in SLE may lead to the discovery of novel therapeutic targets for SLE or maybe other autoimmune diseases.

IL-17A gene transcription is mainly controlled by the retinoic acid receptor–related orphan nuclear receptor-γt (ROR-γt) (9, 10). ROR-γt also promotes the transcription of other T helper (T_h_)17 cell signature genes, including IL-17F, IL-22, and IL-23 receptor (10, 11). Moreover, ROR-γt is required for thymocyte development and lymph node genesis (12). In addition to ROR-γt, IL-17A gene transcription is also regulated by aryl hydrocarbon receptor (AhR) (13, 14). Natural ligand–mediated AhR activation results in an increased T_h_17 population and exacerbated experimental autoimmune encephalomyelitis (EAE) symptoms in mice (15, 16), whereas synthetic ligand–stimulated AhR activation results in an enhanced regulatory T (T_reg_) cell population and ameliorated EAE symptoms in mice (15, 17). In addition to ligand-induced AhR activation, PKC-θ phosphorylates AhR and induces AhR nuclear translocation, leading to induction of IL-17A transcription (18).

MAP kinase kinase kinase kinase 3 [MAP4K3; also named germinal center kinase–like kinase (GLK)] is a member of the MAP4K family (19), which induces activation of the MAPK JNK in epithelial cells (20). GLK induces T-cell activation through phosphorylating and activating PKC-θ (21). Moreover, GLK overexpression occurs in peripheral blood T cells from patients with SLE, RA, or adult-onset Still’s disease; the frequencies of GLK-overexpressing T cells are correlated with autoimmune disease severity (21–23). Recently, using T-cell–specific, GLK-transgenic (Lck-GLK Tg) mice, we found that GLK overexpression selectively induces IL-17A overproduction in T cells, leading to autoimmune responses (18). GLK overexpression induces IL-17A transcription through the IKK-β–mediated ROR-γt Ser-489 phosphorylation and the subsequent AhR–ROR-γt interaction in murine T cells (18). Here, we investigated whether GLK-induced AhR–ROR-γt signaling in T cells is a therapeutic target for human autoimmune diseases such as SLE.

## MATERIALS AND METHODS

### Availability of data and material

The datasets used and analyzed during the current study are available from the corresponding author on reasonable request.

### Human subjects

A total of 87 individuals, including 34 healthy individuals, 48 patients with SLE, and 5 patients with RA, were enrolled in this study. Thirty patients with SLE and 5 patients with RA were referred to the Division of Immunology and Rheumatology at Taichung Veterans General Hospital in Taiwan. Seventeen patients with SLE were referred to the Division of Immunology and Rheumatology at Taipei Veterans General Hospital. One patient with SLE was referred to the Department of Medicine at Chang Gung Memorial Hospital in Taiwan. Written informed consent [approved by the Institutional Review Board at Taichung Veterans General Hospital, Taiwan (C10130B), Taipei Veterans General Hospital (2017-06-003BC), or Chang Gung Memorial Hospital (201701745B0)] was obtained from individual patients before enrollment in this study. All three institutional review boards approved the experiments.

### Mice

Lck-GLK Tg mice with C57BL/6J backgrounds and GLK-deficient mice with C57BL/6J backgrounds were generated as previously described (18, 21). Their respective wild-type littermates with C57BL/6J backgrounds were used as controls. Mice used in this report were either 20-wk-old sex-matched littermates or 8–10-wk-old sex-matched [all female for EAE, all male for collagen-induced arthritis (CIA)] C57BL/6J wild-type mice. All mice used in this study were maintained in temperature-controlled and pathogen-free cages. All animal experiments were performed in the Association for Assessment and Accreditation of Laboratory Animal Care–animal housing facilities at National Health Research Institutes. All mice were used according to the protocols and guidelines approved by the Institutional Animal Care and Use Committee of National Health Research Institutes.

### Antibodies, reagents, and chemicals

**Figure 1:**
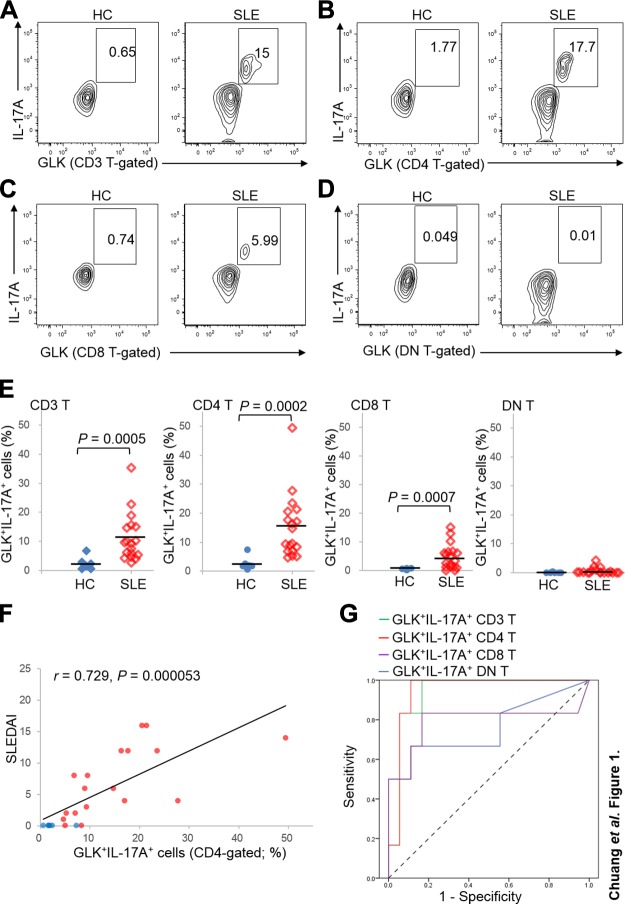
The frequencies of GLK^+^ T_h_17 cells are increased in patients with SLE. *A*–*D*) Flow cytometry analyses of GLK^+^ and IL-17^+^ T cells [CD3-gated, CD3 plus CD4–gated, CD3 plus CD8–gated, or CD3 plus CD4^−^CD8^−^ (DN)–gated] from the PBLs of 6 healthy controls and 18 patients with SLE; the results from a representative healthy control (HC) and a representative patient with SLE (SLEDAI = 12) are shown. *E*) Statistical analyses of GLK^+^IL-17^+^ T cells in the healthy control group and the patient with SLE group are shown. *F*) Positive correlation and significant regression between SLEDAI and the frequencies of GLK^+^IL-17^+^ T cells in the CD4^+^ T subset from all patients with SLE (in red) and healthy controls (in blue). (Pearson correlation coefficient: *r* = 0.729, *P* = 0.000053). *G*) ROC curves of the frequencies of GLK^+^IL-17^+^ cells in T-cell subsets (CD3^+^, CD4^+^, CD8^+^, or CD4^−^CD8^−^ DN subset) for detection of active SLE (SLEDAI ≧ 12). The area under the curve values of individual GLK^+^IL-17^+^ subpopulations: 0.935, *P* = 0.002 (CD3^+^ T cells); 0.944, *P* = 0.001 (CD4^+^ T subset); 0.792, *P* = 0.036 (CD8^+^ T subset); and 0.759, *P* = 0.062 (CD4^−^CD8^−^ DN T subset).

GLK antibody (α-GLK-N) was generated by immunization of rabbits with peptides (murine GLK epitope ^4^GFDLSRRNPQEDFELI^19^, identical to human GLK protein sequences 4–19) and was used for procedures described in **Fig. 1**. The antibody against phosphorylated ROR-γt (Ser-489) was generated by immunization of a rabbit with peptides (murine ROR-γt epitope ^483^FSTDVE[pS]PEGLSK^495^, corresponding to the human ROR-γt protein sequences ^485^FSTETE[pS]PVGLSK^497^). Anti–ROR-γt (clone MABF81) antibody was purchased from MilliporeSigma (Burlington, MA, USA). Anti-AhR (clone RPT9) and anti-vinculin (clone EPR8185) antibodies were purchased from Abcam (Cambridge, MA, USA). The IL-17A ELISA kit was purchased from BioLegend (San Diego, CA, USA). ELISA kits for detection of IL-6, IL-17B, IL-17E, IL-17F, IFN-γ, or TNF-α were purchased from Thermo Fisher Scientific (Waltham, MA, USA). ELISA kits for detection of anti-nuclear antibody, anti–double-stranded DNA (dsDNA) antibody, or rheumatoid factor were purchased from Alpha Diagnostic International (San Antonio, TX, USA). Recombinant proteins of maltose-binding protein–GLK kinase domain fusion proteins and glutathione *S*-transferase (GST)-tagged PKC-θ (K409W) proteins were isolated from *Escherichia coli* (BL21) and then purified by maltose-binding protein or GST pulldown. To remove the maltose-binding protein portion of the GLK kinase domain fusion proteins, the fusion proteins were digested by the protease AcTEV (Thermo Fisher Scientific) and then eluted. The U.S. Food and Drug Administration (FDA)–approved drug library (1200 compounds) was purchased from Prestwick Chemical (Illkirch-Graffenstaden, France). Recombinant proteins of kinase domains of various MAP4Ks were purchased from Promega (Madison, WI, USA).

### *In situ* proximity ligation assay

Proximity ligation assays (PLAs) were performed using the Duolink *In Situ* Red Starter Kit (MilliporeSigma) according to the manufacturer’s instructions (24). Briefly, cells were incubated with rabbit or mouse primary antibodies for the AhR–plus–ROR-γt pair, followed by species-specific secondary antibodies that are conjugated with oligonucleotides (PLA probes). After ligation and amplification reactions for 100 min, the PLA signals from each pair of PLA probes in close proximity (<40 nm) were visualized as individual red dots by a confocal microscope (TCS SP5II; Leica Microsystems, Buffalo Grove, IL, USA). Red dots represent direct interaction signals.

### *In vitro* GLK kinase assay

GLK kinase activity was determined by measuring the rate of ADP production in the *in vitro* kinase reaction using ADP-Glo Kinase Assay Kit (Promega). For the kinase reaction, recombinant proteins of the GLK kinase domain (1 μg) were incubated with recombinant proteins of kinase-dead PKC-θ (K409W; 0.15 μg) plus ATP (0.1 mM) or myelin basic protein (MilliporeSigma) plus ATP (0.1 mM) in kinase buffer [40 mM Tris-HCl (pH 7.5), 20 mM MgCl_2_, 0.1 mg/ml bovine serum albumin, 10 mM DTT, and 0.1 mM Na_3_VO_4_] for 30 min at room temperature.

### Cell-based high‐throughput screening and *in vitro* kinase screening for GLK inhibitors

Cell-based high‐throughput screening was conducted against a representative library of 100,000 molecules at ∼10 μM. The Chinese hamster ovary CHO-K1 (ATCC, CCL-61) cells expressing GLK and NF-κB luciferase (Luc) reporter were used. The reaction volume was 5 μl (500 cells) per well in 1536‐well plates. There was no positive control. This high‐throughput screening campaign was successful, with a determined *Z'* factor of 0.57 ∼ 0.60. However, the cells are very sensitive, and the screening resulted in a total of 17,884 potential hits, which were picked, rescreened, and confirmed. At the end, 1392 compounds were identified for high inhibition (>80%) of the GLK-induced NF-κB luciferase activity of CHO‐GLK‐Luc cells. These 1392 compounds were further screened for the inhibition of GLK kinase activity by *in vitro* kinase assays using the kinase-dead PKC-θ protein as the substrate. From the screening, 2 potential small-molecule GLK inhibitors [half maximal inhibitory concentration (IC_50_) < 10 μM] were identified.

Besides the above 100,000 molecules, 1200 compounds of an FDA-approved drug library were also tested using the same cell-based (>90% inhibition) and *in vitro* screening approaches. From both libraries (101,200 compounds), the most effective inhibitor is verteporfin, which is an FDA-approved drug for macular degeneration of eyes (25). The formula of verteporfin is C_41_H_42_N_4_O_8_. Verteporfin is a light-activated drug for the retinal treatments, although verteporfin was used without any photochemical process in this study.

### Induction of myelin oligodendrocyte peptide–induced EAE

Mice used in EAE experiments were 8–10-wk-old female wild-type C57BL/6J mice. Mice were immunized with 200 μg of myelin oligodendrocyte peptide (MOG) (^35-^MEVGWYRSPFSRVVHLYRNGK^−55^) in complete Freund’s adjuvant [containing 250 μg *Mycobacterium tuberculosis* (Difco, Detroit, MI, USA)] by subcutaneous injection at 2 sites on the back. Mice were also injected with 200 ng pertussis toxin on d 0, 1, and 2. Clinical scores of EAE were monitored daily on a scale of 0–5 as follows: 0, no symptoms; 1, limp tail or 1 hindlimb weakness; 2, limp tail and partial hindlimb paralysis; 3, complete bilateral hindlimb paralysis; 4, complete hindlimb and partial forelimb paralysis; and 5, moribund or death. Mice were treated with verteporfin at a concentration of 0.4 μg/g body weight (0.556 nmol/g) by intravenous injection every 3 d starting at d 1 up to d 22.

### Induction of CIA

At 12 wk of age (d 0), male C57BL/6J mice were intradermally injected in the superficial layer of tail dermis with a total of 100 μl of an emulsion containing the chicken type II chicken collagen or Freund’s complete adjuvant emulsification (MD Biosciences, Oakdale, MN, USA) (23). The same injection was repeated intraperitoneally on d 21. Then, clinical signs of disease were assigned using the following clinical scores: 1, swollen digits; 2, erythema; 3, swollen paw or ankle; 4, loss of function. Mice were treated with verteporfin at a concentration of 0.4 μg/g body weight (0.556 nmol/g) by intravenous injection every 3 d starting from d 21 up to d 36.

### Flow cytometry analyses

For clinical sample analyses, peripheral blood leukocytes (PBLs) were immediately treated with Golgistop (BD Biosciences, San Jose, CA, USA) without any stimulation and then stained with antibodies to surface markers at 25°C. For intracellular staining, PBLs were permeabilized in 200 μl Cytofix or Cytoperm buffer (BD Biosciences) overnight, washed with Perm/Wash buffer (BD Biosciences), and then incubated in antibodies (1:50 dilution) on ice for 2 h. The following antibodies were used for staining: anti–hCD3-PE-Cy7 (SK7), anti–hCD4-Pacific Blue (clone RPA-T4), anti–hCD8-APC-Cy7 (clone SK1), and anti–hIL-17A-Alex647 (clone N49-653). These antibodies were purchased from BD Biosciences. Isotype controls are used as negative controls. Data were collected using FACSCanto II Flow Cytometer (BD Biosciences) and analyzed by FlowJo software (Ashland, OR, USA). Live lymphocytes were first gated based on forward scatter *vs.* side scatter as shown in Supplemental Fig. S1.

### T-cell purification

Primary human T cells were freshly isolated and negatively selected from the PBLs of clinical samples using a cocktail of biotin-conjugated anti-CD19, anti-CD14, and anti-CD11b antibodies (BioLegend) on a magnetic cell separation column (Miltenyi Biotec, Bergisch Gladbach, Germany) (26). In addition, primary murine T cells were negatively selected from the spleens of mice using magnetically coupled antibodies against CD11b, B220, CD49b, CD235, and TER-119 (Miltenyi Biotec) (18).

Immunoblotting analyses and ELISA assays were performed as previously described (21, 27).

### Statistical analyses

All experiments were repeated at least 3 times. Data are presented as means ± sem. The statistical significance between 2 unpaired groups was analyzed using 2-tailed Student’s *t* test. The correlations between the frequencies of GLK^+^IL-17A^+^ T cells and SLE disease activity index (SLEDAI) were evaluated using univariate linear regression analyses. The best cutoff value of the frequencies of GLK^+^IL-17A^+^ T-cell subsets was analyzed by a receiver operating characteristic (ROC) curve. Values of *P* < 0.05 were considered statistically significant. The life-table method was used to plot survival curves of wild-type and GLK-deficient mice. The Wilcoxon pairwise statistic was used to test the significance of the difference between survival curves. All statistical analyses of clinical data were further independently verified by 2 biostatisticians at Taichung Veterans General Hospital.

## RESULTS

### GLK^+^IL-17A^+^ T cells are diagnostic biomarkers for active SLE

To confirm that GLK overexpression induces IL-17A production in T cells of patients with SLE, we characterized T cells from 18 patients with SLE and 6 healthy controls (**Table 1**) using flow cytometry (Supplemental Fig. S1). The flow cytometry data showed that GLK overexpression coexisted exclusively with IL-17A production in T cells of all patients with SLE (Fig. 1*A*). Moreover, the frequencies of GLK^+^IL-17A^+^ cells in CD4^+^ T cells were drastically increased in all 18 patients with SLE compared with those in healthy controls (Fig. 1*B, E*), whereas those in CD8^+^ T cells were modestly increased (Fig. 1*C, E*). In contrast, the frequencies of GLK^+^IL-17A^+^ cells in CD4^−^CD8^−^ [double-negative (DN)] T cells were not significantly increased in the SLE group compared with those in the healthy control group; only 3 patients with SLE showed a very slight increase (Fig. 1*D, E*). These results showed that GLK^+^IL-17A^+^ T cells in patients with SLE were mainly CD4^+^ T cells. Thus, these CD4^+^ T cells were GLK^+^ T_h_17 cells. Moreover, to study whether GLK^+^IL-17A^+^ T-cell population is a useful diagnostic biomarker for active SLE (SLEDAI ≧ 12), the diagnostic utility of the frequencies of GLK^+^IL-17A^+^ T-cell subsets were analyzed by liner regression and ROC curve analyses (Fig. 1*F, G*). The frequencies of GLK^+^IL-17A^+^ cells in CD4^+^ T cells were correlated (*r* = 0.729, *P* = 0.00053) with SLEDAI (Fig. 1*F*). According to the coordinates of the ROC curve, the best cutoff value for the frequencies of GLK^+^IL-17A^+^ cells in CD4^+^ T-cell population was 15.5%. Moreover, the frequencies of GLK^+^IL-17A^+^ cells in CD4^+^ T cells had a sensitivity of 100% and a specificity of 88.9% for identifying active patients with SLE (Fig. 1*G*). Thus, GLK^+^ T_h_17 cell is a diagnostic biomarker for active SLE. Notably, the area under the curve value (0.939, *P* < 0.001) of GLK^+^IL-17A^+^ frequencies in CD4^+^ T cells was significantly more than that in CD8^+^ or DN T cells. Collectively, these results suggest that GLK overexpression in T cells induces IL-17A production and pathogenic T_h_17 cells, contributing to the pathogenesis of a distinct subpopulation of patients with SLE.

**TABLE 1:** Demographic and disease characteristics of patients with SLE and healthy controls

Characteristic	SLE (*n* = 18)	HC (*n* = 6)
Age at study entry (yr)	38.2 ± 16.0	31.0 ± 6.0
Sex [female (%)]	17 (94.4)	6 (100)
Disease duration (yr)	8.0 ± 5.8	NA
SLEDAI	7.1 ± 5.4	NA
Fever (%)	1 (5.6)	NA
Arthritis (%)	7 (38.9)	NA
Nephritis (%)	10 (55.6)	NA
Malar rash (%)	7 (38.9)	NA
Serositis (%)	2 (11.1)	NA
Neurologic disorders (%)	5 (27.8)	NA
WBC (/mm^3^)	6322 ± 2364	NA
HgB (g/dl)	12.3 ± 1.9	NA
Platelet (1000/mm^3^)	271.5 ± 90.6	NA
Creatinine (mg/dl)	0.86 ± 0.2	NA
Anti-dsDNA antibody (WHO U/ml)	213.7 ± 185.7	NA
C3 (mg/dl)	84.8 ± 31.1	NA
C4 (mg/dl)	20.9 ± 14.1	NA

Demographic characteristics were determined only at the beginning of SLE diagnosis. Durations refer to the length of the disease time after SLE diagnosis. Plus-minus values are means ± sd. C3/4, complement component 3/4; HC, healthy controls; HgB, hemoglobin; NA, not applicable; WBC, white blood cell; WHO, World Health Organization.

### GLK induces ROR-γt Ser-489 phosphorylation and the AhR–ROR-γt complex in human autoimmune patient T cells

**Figure 2:**
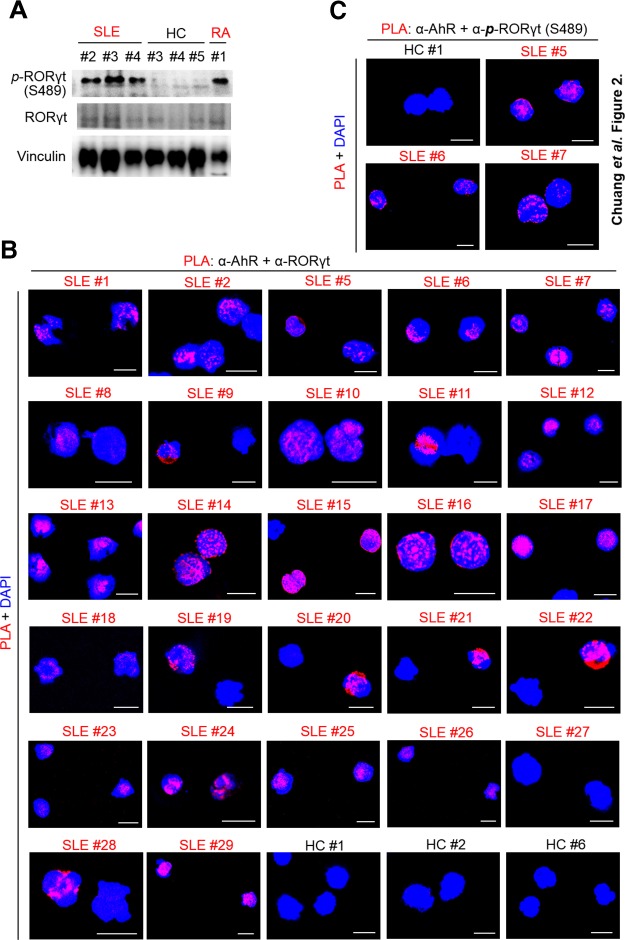
Phosphorylated ROR-γt interacts with AhR in human autoimmune T cells. *A*) Immunoblotting analyses of phosphorylated ROR-γt (Ser-489) and ROR-γt in peripheral blood T cells freshly isolated from 3 patients with SLE, 1 patient with RA, and 3 healthy controls (HCs). *B*) Confocal microscopy analyses of PLA for the interaction between AhR and ROR-γt in peripheral blood T cells, which were freshly isolated from 27 patients with SLE and 23 healthy controls (3 representative healthy controls are shown; 20 additional healthy controls and 3 patients with RA are shown in Supplemental Fig. S2). *C*) Confocal microscopy analyses of PLA for the interaction between endogenous AhR and phosphorylated ROR-γt proteins in peripheral blood T cells (3 representative patients with SLE and 1 healthy control are shown; 22 additional patients with SLE, 20 additional healthy controls, and 2 patients with RA are shown in Supplemental Fig. S3). For PLA, red dots represent direct interaction signals. T-cell nucleus was stained with DAPI (blue color). Original magnification, ×630. Scale bar, 10 μm.

Because we found that GLK overexpression induces ROR-γt Ser-489 phosphorylation and the AhR–ROR-γt complex (18), we further studied whether ROR-γt Ser-489 phosphorylation and the AhR–ROR-γt complex occur in T cells of human patients with SLE using immunoblotting and *in situ* PLA. Immunoblotting data showed that ROR-γt Ser-489 phosphorylation was drastically enhanced in unstimulated peripheral blood T cells freshly isolated from patients with SLE and RA (**Fig. 2*A***). Furthermore, unstimulated peripheral blood T cells from all but 1 (26/27) of the human patients with SLE displayed numerous PLA interaction signals between AhR and ROR-γt, whereas T cells from healthy controls did not show any interaction (Fig. 2*B* and Supplemental Fig. S2). Consistently, the PLA assays using both anti–phosphorylated ROR-γt (Ser-489) antibody and anti-AhR antibody also showed many PLA interaction signals between phosphorylated ROR-γt (Ser-489) and AhR (Fig. 2*C* and Supplemental Fig. S3). Many PLA interaction signals between AhR and either ROR-γt or phosphorylated ROR-γt (Ser-489) were also detected in T cells from patients with RA (Supplemental Figs. S2 and S3). Thus, the GLK–AhR–ROR-γt signaling is indeed induced in human SLE T cells.

### Identification of the small-molecule inhibitor verteporfin for GLK-induced AhR–ROR-γt complex in human autoimmune patient T cells

**Figure 3:**
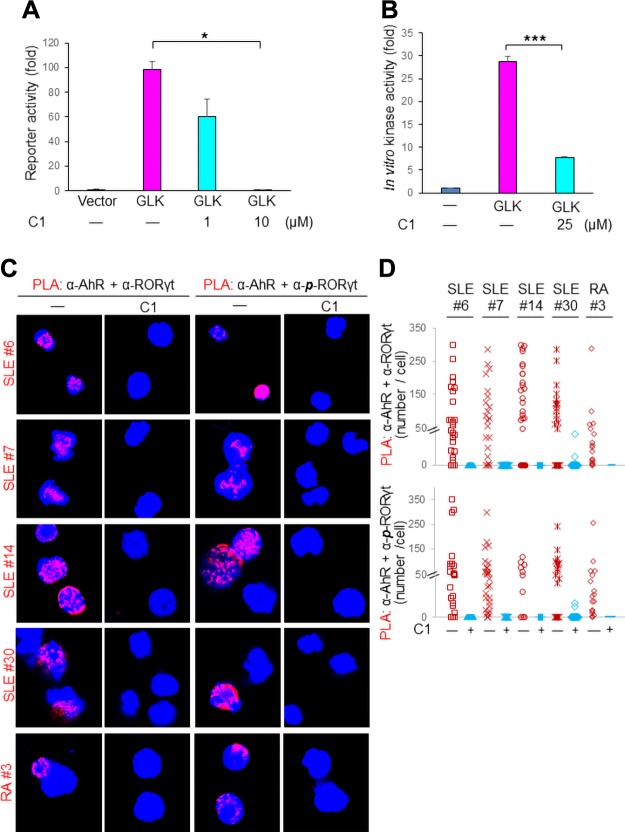
Verteporfin (C1) blocks the AhR–ROR-γt complex in human autoimmune T cells. *A*) Luciferase activity of NF-κB–driven reporter assays in stable GLK-transfected CHO-K1 cells that were treated with or without C1 under the indicated concentration. The fold changes are presented relative to the value of vector control. *B*) Recombinant proteins of GLK kinase domain plus recombinant proteins of GST-tagged kinase-dead PKC-θ (K409W) were subjected to *in vitro* kinase assays in the presence or absence of C1. The fold changes are presented relative to the value of the substrate alone as a control. Means ± sd of are shown. *C*) The AhR–ROR-γt complex was inhibited by C1. Confocal microscopy analyses of PLA for the interaction between AhR and either ROR-γt or phosphorylated ROR-γt (S489) in peripheral blood T cells treated with C1 (5 μM) for 30 min. The T cells were freshly isolated from 4 patients with SLE and 1 patient with RA. Original magnification, ×630. *D*) Quantification of the PLA signals shown in panel *C*. *P < 0.05, ***P < 0.001 (2-tailed Student’s *t* test).

After verification of the induction of GLK–AhR–ROR-γt–IL-17A signaling in human SLE T cells, we next studied whether the GLK–AhR–ROR-γt signaling is a therapeutic target for autoimmune diseases. Because there are no known commercial GLK inhibitors, we performed high-throughput screening of 101,200 compounds to identify GLK inhibitors using both cell-based luciferase reporter assays and *in vitro* kinase assays. From the screening, one compound, verteporfin, efficiently inhibited both GLK signaling in cell lines (**Fig. 3*A***) and GLK kinase activity *in vitro* (Fig. 3*B*). The IC_50_ of verteporfin for GLK kinase activity *in vitro* was 1.15 nM (**Table 2**), which was drastically lower than the concentration (25 μM) used for the initial high-throughput screening. Moreover, the IC_50_ values of verteporfin for MAP4K1 [also named hematopoietic progenitor kinase 1 (HPK1)] and MAP4K4 [also named HPK1/germinal center kinase-like kinase (HGK)] kinase activity *in vitro* were 7.91 and 4.92 nM, respectively (Table 2). Nevertheless, the IC_50_ of verteporfin for MAP4K3-GLK was the lowest one. We next tested whether verteporfin suppresses AhR–ROR-γt complex formation. Peripheral blood T cells freshly isolated from patients with SLE or RA were treated with verteporfin for 30 min and subjected to *in situ* PLA. We found that verteporfin indeed blocked both the AhR–ROR-γt complex and the AhR–phosphorylated ROR-γt complex in peripheral blood T cells from patients with SLE and RA (Fig. 3*C*). Quantification of the PLA signals showed that the verteporfin treatment significantly inhibited the GLK-induced AhR–ROR-γt complex and AhR–phosphorylated ROR-γt complex (Fig. 3*D*).

**TABLE 2:** The IC_50_ of verteporfin for MAP4Ks

IC_50_	GLK (MAP4K3)	HPK1 (MAP4K1)	GCK (MAP4K2)	HGK (MAP4K4)	KHS (MAP4K5)
verteporfin	1.15 nM	7.91 nM	10.29 nM	4.92 nM	43.88 nM

Inhibition of kinase activity of GLK (MAP4K3) or other MAP4Ks was determined by *in vitro* kinase assays using myelin basic protein as a substrate. Recombinant proteins of kinase domains of various MAP4Ks were used.

### Verteporfin suppresses IL-17A production and autoimmune responses in mice

**Figure 4:**
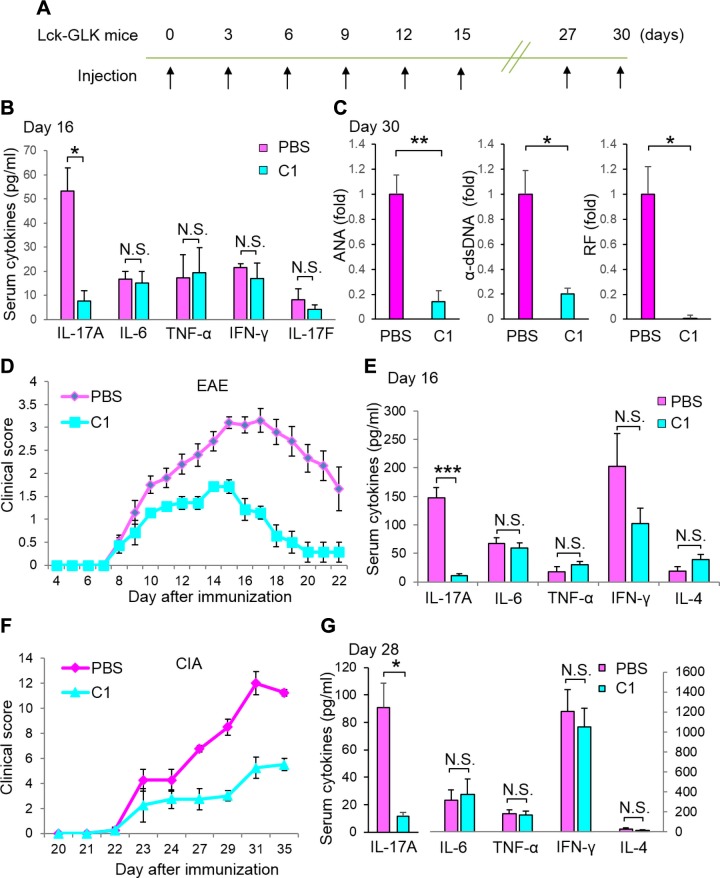
Verteporfin (C1) suppresses IL-17 production and autoimmune responses in mice. *A*–*C*) Effect of C1 administration (0.4 μg/g body weight every 3 d for a period of 30 d) on serum IL-17A and autoantibodies in 20-wk-old Lck-GLK Tg mice. *B*) ELISA of serum IL-17A, IL-6, TNF-α, IFN-γ, and IL-17F in Lck-GLK Tg mice treated with C1 or PBS for 16 d. *C*) ELISA of serum autoantibodies in Lck-GLK Tg mice treated with C1 or PBS for 30 d. Means ± sem are shown; *n* = 4/group. *D*) EAE induction in wild-type C57BL/6J mice treated with PBS or C1 (0.4 μg/g body weight every 3 d). Clinical scores are presented on a scale of 1–5. PBS group, *n* = 10; C1 group, *n* = 7. *E*) ELISA of IL-17A in the sera from MOG-immunized mice at day 16. *F*) Induction of CIA in wild-type C57BL/6J mice treated with C1 (0.4 μg/g body weight every 3 d) or PBS. Clinical scores are presented on a scale of 1–16; *n* = 4/group. *G*) ELISA of serum IL-17A in collagen-immunized mice at d 28. Data shown are representatives of 3 independent experiments. α-dsDNA, anti-dsDNA antibody; ANA, anti-nuclear antibody; N.S., not significant; RF, rheumatoid factor. Means ± sem are shown. *P < 0.05, **P < 0.01, ***P < 0.001 (2-tailed Student’s *t* test).

The GLK-induced AhR–ROR-γt complex controls IL-17A production and autoimmune responses in mice (18); therefore, we studied whether verteporfin also inhibits IL-17A production and autoimmune responses in Lck-GLK Tg mice. Lck-GLK Tg mice were administered verteporfin (0.4 μg/g body weight) by intravenous injection every 3 d for a period of 30 d (**Fig. 4*A***). The induction of serum IL-17A in Lck-GLK Tg mice was significantly decreased by the verteporfin treatment compared with that of the PBS treatment for 16 d (Fig. 4*B*), whereas the serum levels of IL-6, TNF-α, IFN-γ, and IL-17F in Lck-GLK mice were not significantly changed. Moreover, overproduction of autoantibodies (anti-nuclear antibody, anti-dsDNA antibody, and rheumatoid factor) in Lck-GLK Tg mice was also abolished by the verteporfin treatment for 30 d (Fig. 4*C*). These results support that GLK overexpression induces IL-17A overproduction and autoantibody production. To further verify that verteporfin is also effective in IL-17A–mediated animal models, we treated wild-type mice with verteporfin during induction of EAE and CIA. The verteporfin treatment (0.4 μg/g body weight) significantly suppressed the disease’s severity and serum IL-17A levels of mice during the EAE (Fig. 4*D*) or CIA induction (Fig. 4*F*). Similar to the data derived from Lck-GLK mice, the serum levels of IL-6, TNF-α, IFN-γ, and IL-4 in the diseased mice were not significantly decreased by the verteporfin treatment (Fig. 4*E, G*). The data showed that the verteporfin treatment preferentially inhibited IL-17A overproduction in all 3 autoimmune disease models. In addition, the verteporfin treatment inhibited both T_h_17 differentiation and IL-17A production from *in vitr*o differentiated T_h_17 cells (Supplemental Fig. S5). These data suggest that verteporfin is a potential therapeutic for IL-17A–mediated autoimmune diseases.

### The inhibitor of GLK signaling blocks IL-17A production of human T cells

**Figure 5:**
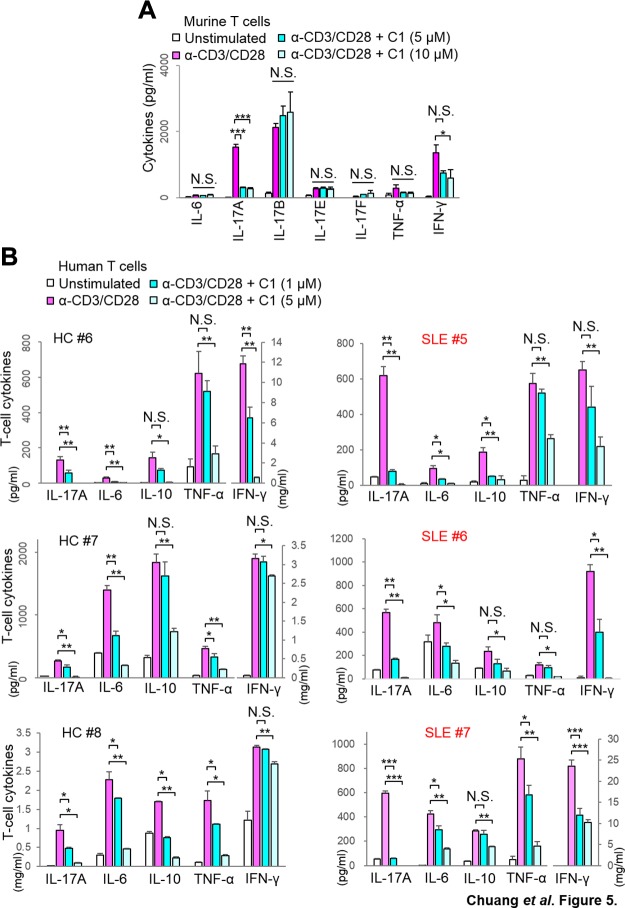
Verteporfin (C1) blocks IL-17 production of human T cells. *A*) ELISA of various cytokines in supernatants of murine primary T cells stimulated with anti-CD3 or CD28 and cotreated with C1 (5 or 10 μM) for 3 d. Means ± sd are shown; *n* = 3/group. *B*) ELISA of various cytokines in supernatants of human T cells. T cells were stimulated with anti-CD3 or CD28 and cotreated with C1 (1 or 5 μM) for 3 d. HC, healthy control; N.S., not significant. Means ± sd are shown. *P < 0.05, **P < 0.01, ***P < 0.001 (2-tailed Student’s *t* test).

We further studied whether IL-17A production is abolished by verteporfin in human and murine T cells. We first tested the inhibitory effect of verteporfin in murine T cells stimulated with T-cell receptor (TCR) signaling (anti-CD3 plus anti-CD28 antibodies). The verteporfin treatment for 3 d did not enhance cell death of murine primary T cells (Supplemental Fig. S4). We found that TCR-induced IL-17A production of murine T cells was drastically abolished by the verteporfin treatment, whereas TCR-induced IL-17B, IL-17E, and IL-17F levels were not significantly decreased (**Fig. 5*A***). TCR-induced TNF-α levels were slightly but not significantly reduced by the verteporfin treatment. TCR-induced IFN-γ levels were significantly inhibited by the high-dose (10 μM) verteporfin treatment (Fig. 5*A*). This reduction is likely due to the inhibition of NF-κB activation (18) by the verteporfin treatment. This notion is supported by our published results that TCR-induced TNF-α and IFN-γ levels in T cells are inhibited by IKK-β conditional knockout (18). Next, we studied whether verteporfin attenuates IL-17A production using human peripheral blood T cells with stimulation. The levels of T-cell–secreted IL-17A in unstimulated T cells from patients with SLE and RA were enhanced compared with those from healthy controls (Fig. 5*B*). To study whether verteporfin preferentially inhibits IL-17A production, T cells from human subjects were stimulated with anti-CD3 plus anti-CD28 antibodies to induce production of IL-17A and other cytokines. IL-17A production was significantly reduced by the verteporfin treatment of peripheral blood T cells from patients with SLE and RA as well as from healthy controls (Fig. 5*B* and Supplemental Fig. S6*A*). Notably, inhibition of IL-17A (∼75%) by the verteporfin treatment (1 μM) in patients with SLE or RA was more efficient than that (∼48%) in healthy controls (Supplemental Fig. S6*B*). GLK overexpression in T cells selectively induces IL-17A production in Lck-GLK Tg mice (18). The previous finding by Chuang *et al*. (18) and the data in this report suggest that verteporfin (both high-dose and low-dose) efficiently suppresses GLK overexpression–induced IL-17A overproduction in inflammatory T cells from patients with SLE or RA. Unlike the efficient inhibition of IL-17A production by the low-dose (1 μM) verteporfin treatment, the production of IL-6, IL-10, TNF-α, and IFN-γ in T cells from patients with SLE or RA was modestly inhibited by the low-dose (1 μM) verteporfin treatment (Fig. 5*B*). Under a high-dose (5 μM) verteporfin treatment, the production of IL-17A, IL-6, IL-10, TNF-α, and IFN-γ was significantly inhibited in T cells of both healthy controls and patients with SLE (Fig. 5*B*). The inhibition of the production of other cytokines by the high-dose but not low-dose verteporfin treatment may be due to a requirement of higher concentration of verteporfin for an efficient inhibition of NF-κB activation. Collectively, these data suggest that verteporfin efficiently blocks IL-17A overproduction by inhibiting the GLK–AhR–ROR-γt pathway.

### GLK-deficient mice display prolonged lifespan

**Figure 6:**
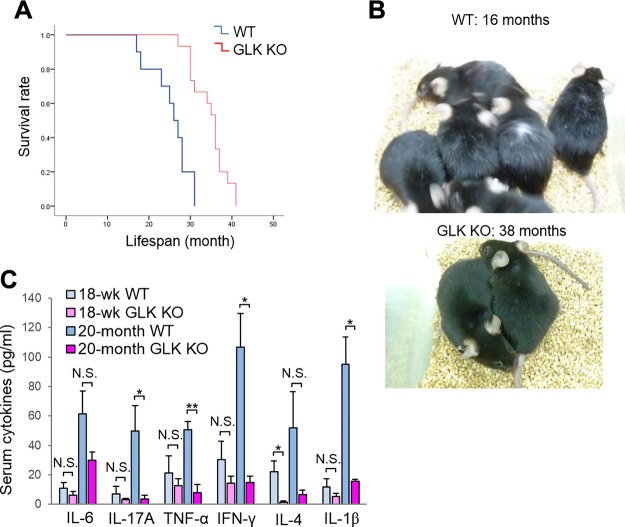
GLK-deficient mice display an increased lifespan. *A*) Survival curves of wild-type or GLK-deficient mice were calculated by the life-table method. Shown are survival plots representing pooled data from 10 wild-type mice and 15 GLK-deficient mice. GLK-deficient mice exhibited a significant extension of lifespan relative to wild-type mice (Wilcoxon test, *P* = 0.001). *B*) 16-mo-old wild-type mice displayed gray hair, whereas 38-mo-old GLK-deficient mice still displayed healthy hair. *C*) ELISA of various cytokines in the sera of 4.5-mo-old wild-type, 20-mo-old wild-type, or 20-mo-old GLK-deficient mice. Means ± sem are shown; *n* = 4/group. WT, wild-type mice; GLK KO, GLK-deficient mice; N.S., not significant. *P < 0.05, **P < 0.01 (2-tailed Student’s *t* test).

To study whether inhibition of GLK may result in any spontaneous diseases, we monitored both wild-type and GLK-deficient mice (21) during their lifetimes. Surprisingly, GLK-deficient mice displayed a significant extension of lifespan compared with wild-type mice (**Fig. 6*A***). The oldest GLK-deficient mouse was 40.8 mo of age. The aged GLK-deficient mice (26.0–40.8 mo) did not develop any tumor or discernible diseases. Interestingly, the aged GLK-deficient mice still displayed healthy hair, whereas the aged wild-type mice displayed gray hair (Fig. 6*B*). A previous publication indicates that T-cell–mediated immune responses, including antigen-induced IFN-γ, IL-2, and IL-17A production, are decreased in GLK-deficient T cells (21). Moreover, T_h_1, T_h_2, and T_h_17 differentiation is reduced by GLK deficiency (21). GLK-deficient mice are also resistant to the induction of MOG-induced EAE (21). The longevity of GLK-deficient mice may be due to the inhibition of inflammatory responses by GLK deficiency. This notion is supported by the data indicating that serum levels of the proinflammatory cytokine IL-6, IL-17A, TNFα, IFN-γ, and IL-1β in 20-mo-old GLK-deficient mice were drastically decreased compared with those of age-matched wild-type mice (Fig. 6*C*). Besides T_h_1 cytokine (IFN-γ) and T_h_17 cytokine (IL-17A) levels, T_h_2 cytokine (IL-4) levels were also decreased in the sera of aged GLK-deficient mice (Fig. 6*C*). The data suggest that inhibition of GLK would not lead to any adverse effects but instead may prevent age-related inflammation.

## DISCUSSION

GLK overexpression in T cells is involved in the pathogenesis of several human autoimmune diseases, including SLE (21), RA (23), and adult-onset Still’s disease (22). The GLK–AhR–ROR-γt–IL-17A signaling identified using murine T cells (18) is further verified in this study using purified T cells from human patients with SLE. Moreover, verteporfin suppresses the GLK-induced AhR–ROR-γt complex and IL-17A production in human patients with SLE T cells as well as the disease severity of 3 autoimmune animal models. GLK overexpression in Lck-GLK Tg T cells or T cells of patients with SLE selectively induces IL-17A production; however, GLK deficiency (mimicked by high-dose verteporfin treatment) blocks the production of both NF-κB–induced T_h_1 or T_h_2 cytokines and AhR–ROR-γt–induced IL-17A. Furthermore, GLK-deficient mice display longer lifespans than those of wild-type mice, suggesting that inhibition of GLK may be a feasible approach for treating human diseases such as SLE. Thus, GLK, GLK-induced signaling molecules, or the GLK-induced AhR–ROR-γt complex can be a promising therapeutic target for patients with GLK^high^IL-17A^high^ SLE.

ROR-γt inhibitors have been considered for treatment of autoimmune diseases and inflammation-associated diseases; however, ROR-γt inhibitors also induce side effects in animal studies due to the impairment of thymocyte development and dysregulation of other ROR-γt–regulated genes (28, 29). Moreover, adult ROR-γt–deficient mice develop lymphoblastic lymphoma (30). Similarly, AhR regulates different immune responses through activating other AhR-regulated genes. For example, AhR differentially regulates the activation of T_reg_ or inflammatory T_h_17 cells under different stimuli (15, 16). Thus, inhibition of either ROR-γt or AhR activity for treating human disease appears to be very challenging. In contrast, the GLK-induced AhR–phosphorylated ROR-γt complex selectively stimulates IL-17A gene transcription (18). Thus, inhibition of the physical interaction between AhR and phosphorylated ROR-γt would abolish IL-17A production (Fig. 5*A*) without affecting the production of other ROR-γt– or AhR-regulated genes, including cytokines.

One previous publication by Crispín et al. (31) reported that IL-17A–producing DN T cells, instead of CD4^+^ T cells, are significantly increased in patients with SLE. This report is not supported by several subsequent publications, which reported that the T_h_17 population is increased in patients with SLE (2–5). Our data further showed that the frequencies of pathogenic GLK^high^ T_h_17 cells are correlated with SLEDAI. Given that GLK and IL-17A are exclusively coexpressed in the T cells of a subpopulation of patients with SLE and that Lck-GLK Tg mice spontaneously develop autoimmune phenotypes, GLK overexpression in T cells may lead to T_h_17-mediated pathogenesis of SLE. We propose that IL-17A blockade may be useful for treating the GLK-high or T_h_17-high subpopulation of patients with SLE. Thus, using GLK^+^ T_h_17 cell as a biomarker will help with selection of patients in this subpopulation (GLK^high^IL-17A^high^) that are likely responsive to IL-17A blockade or GLK inhibitors, leading to precision medicine for SLE.

IL-17A blockade using anti-IL-17A monoclonal antibody shows therapeutic effects on autoimmune disease (7, 8); however, the protein drug is much more costly than the small-molecule compound inhibitor. In this study, we found that the FDA-approved small-molecule verteporfin inhibits GLK kinase activity as well as inhibits IL-17A production in human autoimmune patient T cells and attenuates autoimmune responses in 3 mouse models. Thus, the small-molecule compound verteporfin could be a cost-effective therapeutic agent for autoimmune disease. In addition, a higher concentration of verteporfin also inhibits kinase activity of MAP4K1-HPK1 and MAP4K4-HGK *in vitro*. Notably, MAP4K1-HPK1 is a negative regulator of TCR signaling (32); therefore, inhibition of MAP4K1-HPK1 would result in overproduction of multiple cytokines. MAP4K4-HGK plays an inhibitory role in activation of IL-6^+^IL-17^+^ T cells (27); therefore, inhibition of MAP4K4-HGK would result in overproduction of IL-6 and IL-17. According to the aforementioned reasons, inhibition of IL-17A production or T_h_17-mediated autoimmune responses by the verteporfin treatment would not due to inactivation of HPK1 or HGK. There are 3 potential mechanisms of GLK inhibition by verteporfin. First, verteporfin may bind to the ATP-binding site of GLK, resulting in attenuation of GLK kinase activity. Second, verteporfin may bind to the interface of the GLK homodimer, leading to disruption of GLK dimerization and subsequent reduction of GLK autophosphorylation or autoactivation. Lastly, verteporfin may inhibit GLK kinase activity by inducing a conformational change of GLK protein.

Our findings suggest that GLK-induced AhR–ROR-γt complex is a biomarker and therapeutic target for IL-17A–mediated autoimmune disease. In addition, verteporfin may be a potential treatment for IL-17A–mediated autoimmune disease.

## Supplementary Material

This article includes supplemental data. Please visit *http://www.fasebj.org* to obtain this information.

## Abbreviations

AhR: aryl hydrocarbon receptor
CIA: collagen-induced arthritis
CHO: Chinese hamster ovary
DN: double negative
dsDNA: double-stranded DNA
EAE: experimental autoimmune encephalomyelitis
FDA: Food and Drug Administration
GLK: germinal center kinase–like kinase
GST: glutathione *S*-transferases
HGK: HPK1/germinal center kinase-like kinase
HPK1: hematopoietic progenitor kinase 1
IC_50_: half maximal inhibitory concentration
Lck-GLK Tg: T-cell–specific, GLK-transgenic
MAP4K3: MAP kinase kinase kinase kinase 3
MOG: myelin oligodendrocyte peptide
PBL: peripheral blood leukocyte
PLA: proximity ligation assay
RA: rheumatoid arthritis
ROC: receiver operating characteristic
ROR-γt: retinoic acid receptor–related orphan nuclear receptor-γt
SLE: systemic lupus erythematosus
SLEDAI: SLE disease activity index
TCR: T-cell receptor
T_h_: T helper
