# Supplementary material for: AhR–ROR-γt complex is a therapeutic target for MAP4K3/GLKhighIL-17Ahigh subpopulation of systemic lupus erythematosus
Source: FASEB J. 2019 Aug 1;33(10):11469–80. doi: 10.1096/fj.201900105RR (PMC6766655; doi:10.1096/fj.201900105RR)
Supplement: Supplementary file 1 [file fj.201900105RR.sd1.docx]

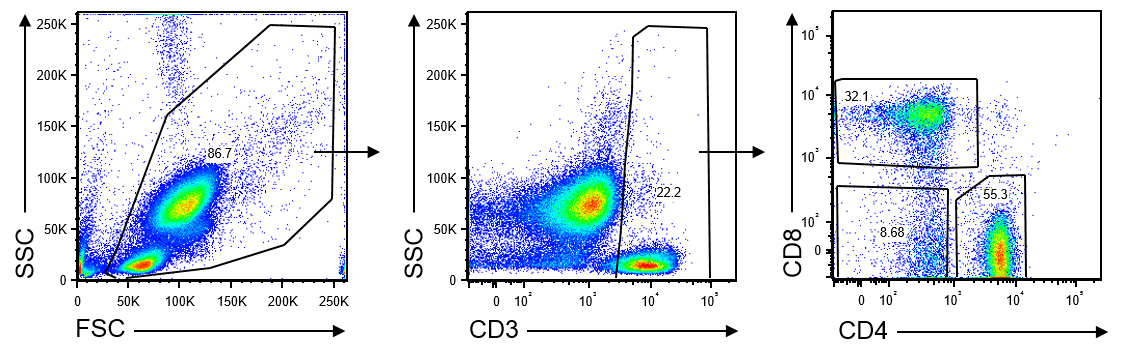


**Supplementary Figure 1. Gating approach of clinical sample analyses using flow cytometry.** Peripheral blood leukocytes from human individuals were analyzed by flow cytometry. Plots are gated on CD3^+^ T cells and then gated on CD4^+^, CD8^+^, or CD4^-^CD8^-^ (double-negative) T cells. Numbers indicate the percentage of cells for individual subpopulations.


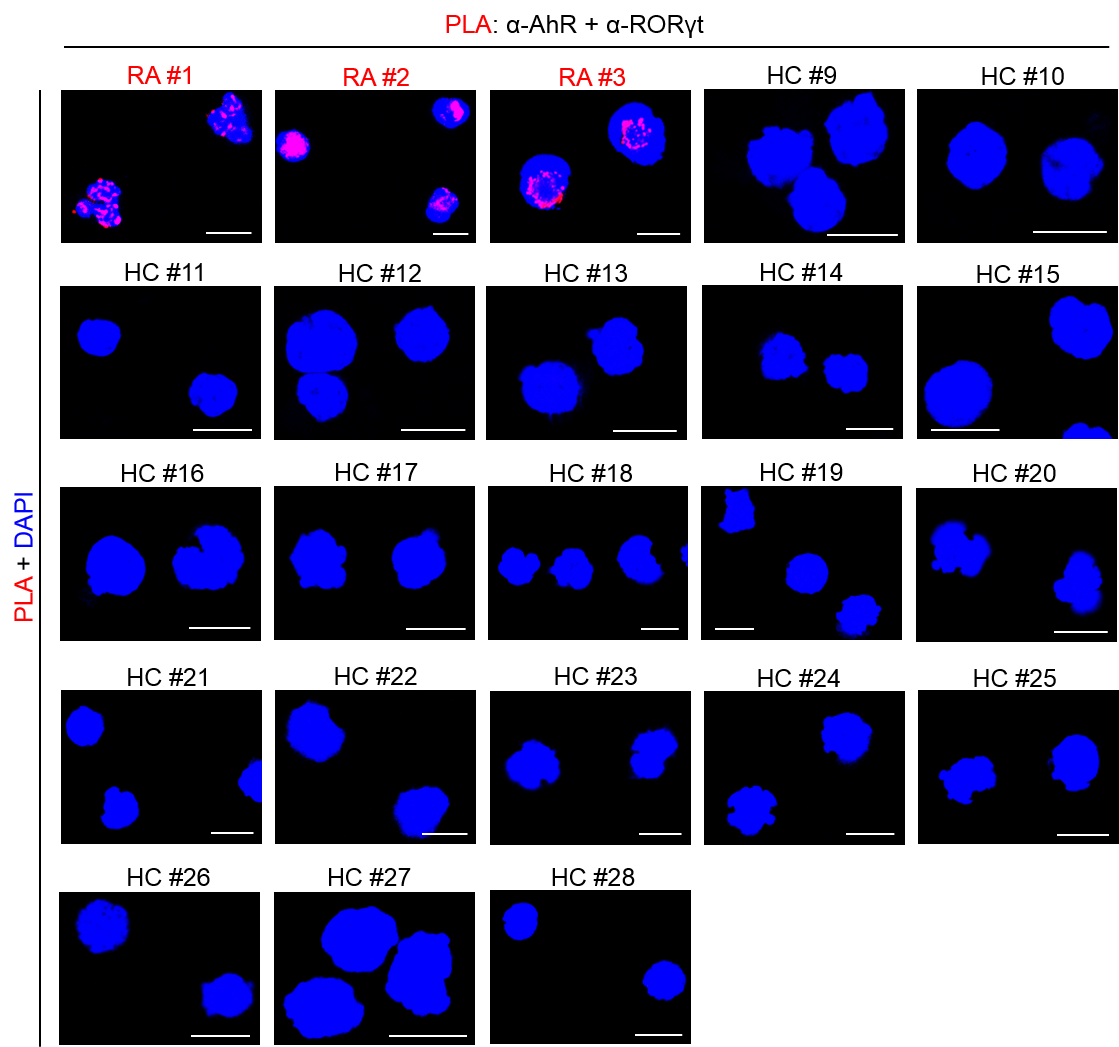


**Supplementary Figure 2. RORγt interacts with AhR in human autoimmune T cells.** Confocal microscopy analyses of proximity ligation assays (PLA) for the interaction between endogenous AhR and RORγt proteins in peripheral blood T cells, which were freshly isolated from 3 RA patients and 20 healthy controls. For PLA, red dots represent direct interaction signals. T-cell nucleus was stained with DAPI (blue color). Original magnification, × 630; bar, 10 μm.


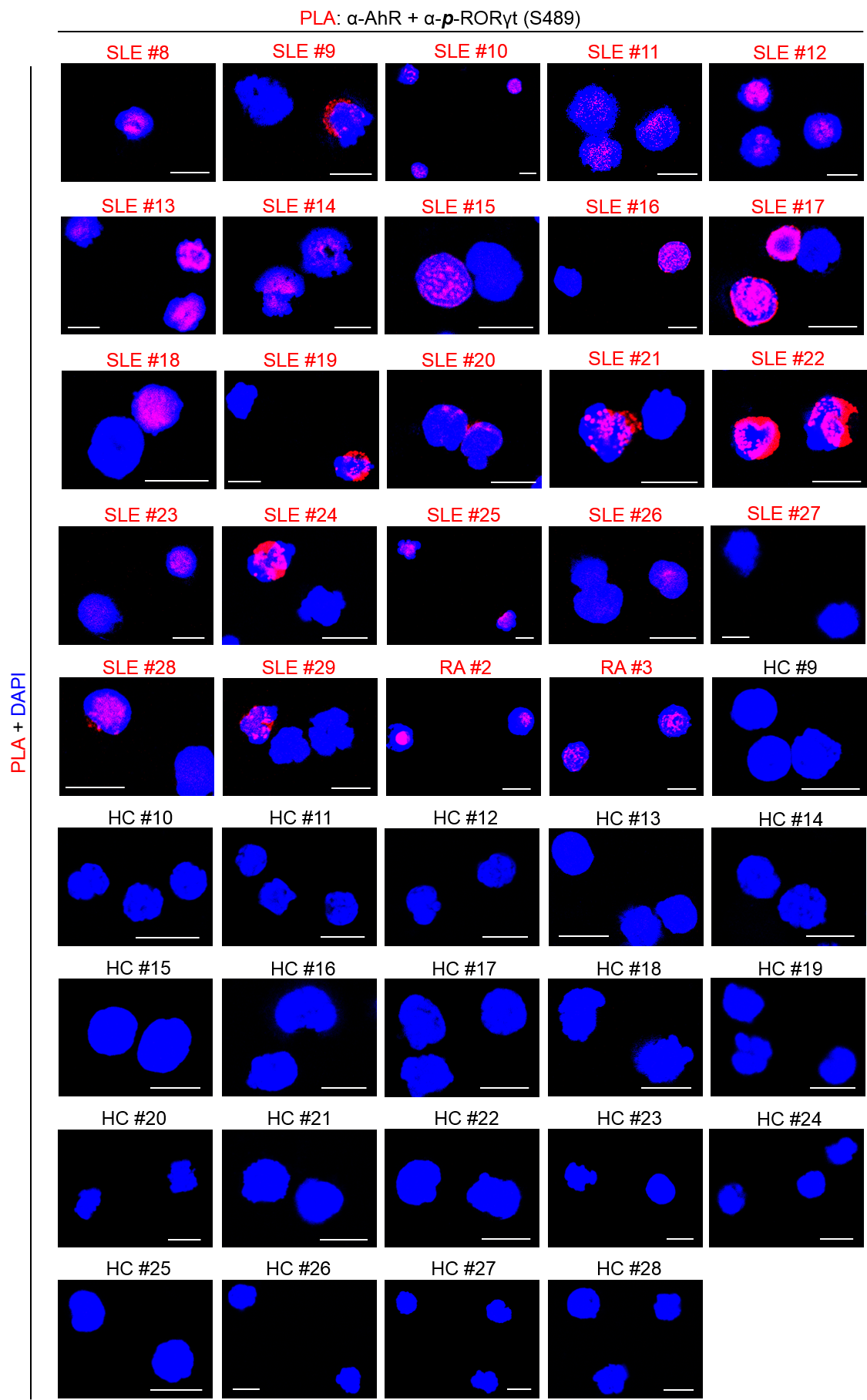


**Supplementary Figure 3. Phosphorylated RORγt interacts with AhR in human autoimmune T cells.** Confocal microscopy analyses of proximity ligation assays (PLA) for the interaction between endogenous AhR and phosphorylated RORγt proteins in peripheral blood T cells from 22 SLE patients, 2 RA patients, 20 healthy controls. SLE, systemic lupus erythematosus; RA, rheumatoid arthritis. For PLA, red dots represent direct interaction signals. T-cell nucleus was stained with DAPI (blue color). Original magnification, × 630; bar, 10 μm.


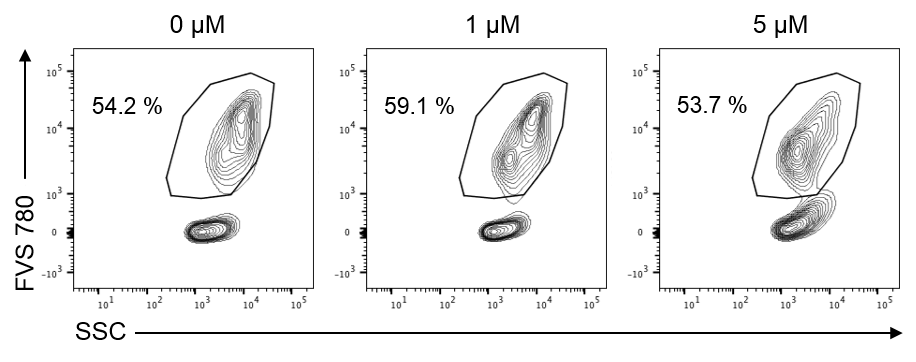


**Supplementary Figure 4. Verteporfin does not induce cell death of murine T cells.** Murine primary T cells were stimulated with anti-CD3/CD28 and co-treated with verteporfin (C1) (1 or 5 μM) for 3 days. Dead T cells were labeled with Fixable Viability Stain 780 (FVS 780; BD Biosciences) and then determined by flow cytometry. Data shown are representatives of four independent experiments.


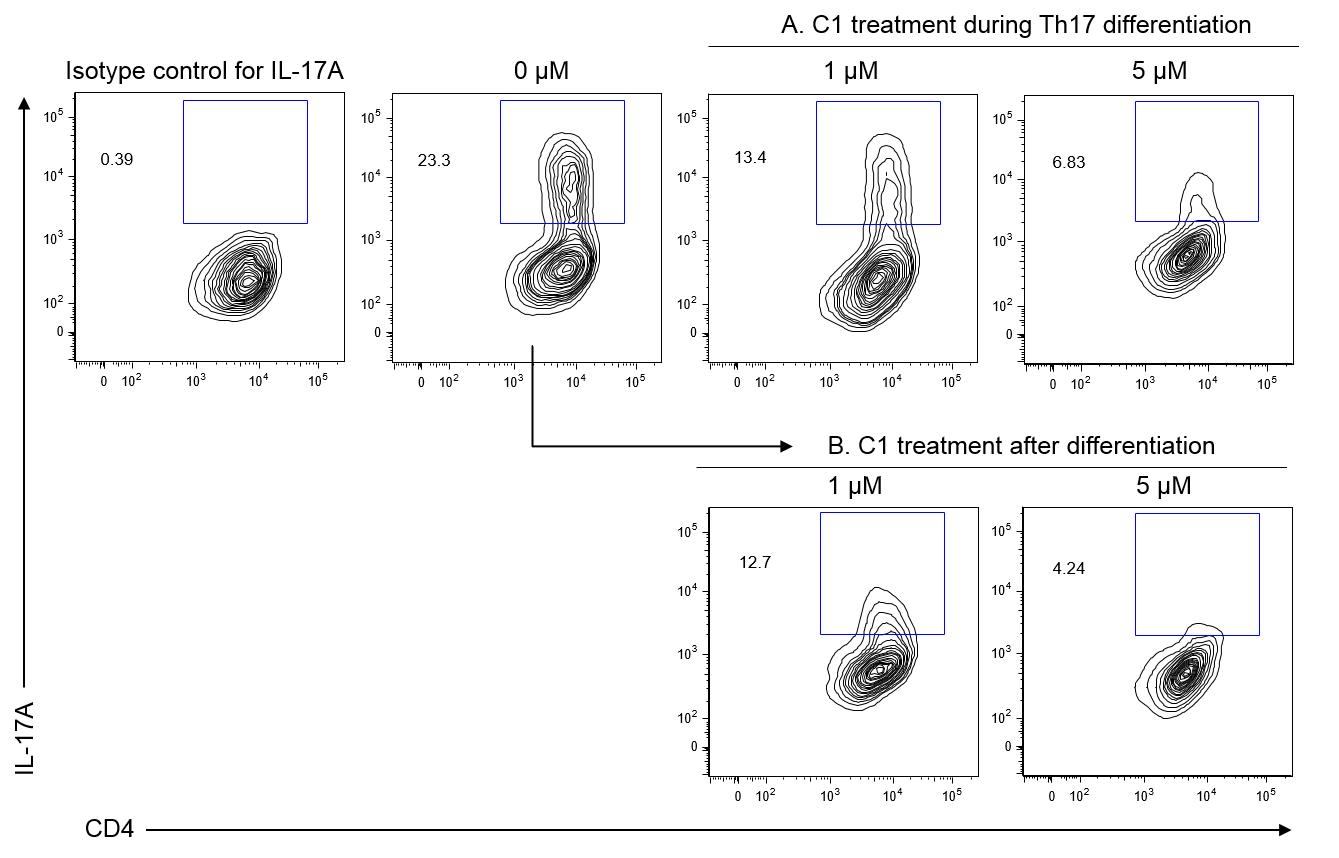


**Supplementary Figure 5. Verteporfin inhibits both Th17 differentiation and IL-17A production from *in vitro* differentiated Th17 cells. (A)** Flow cytometry of IL-17A-producing CD4^+^ T cells. Splenic T cells were co-treated with verteporfin (C1) (1 or 5 μM) during Th17 differentiation *in vitro* (for 3 days). **(B)** Flow cytometry of IL-17A-producing CD4^+^ T cells. Murine *in vitro* differentiated Th17 cells were stimulated with PMA plus ionomycin and co-treated with verteporfin (C1) (1 or 5 μM) for 30 min. Data shown are representatives of two independent experiments.


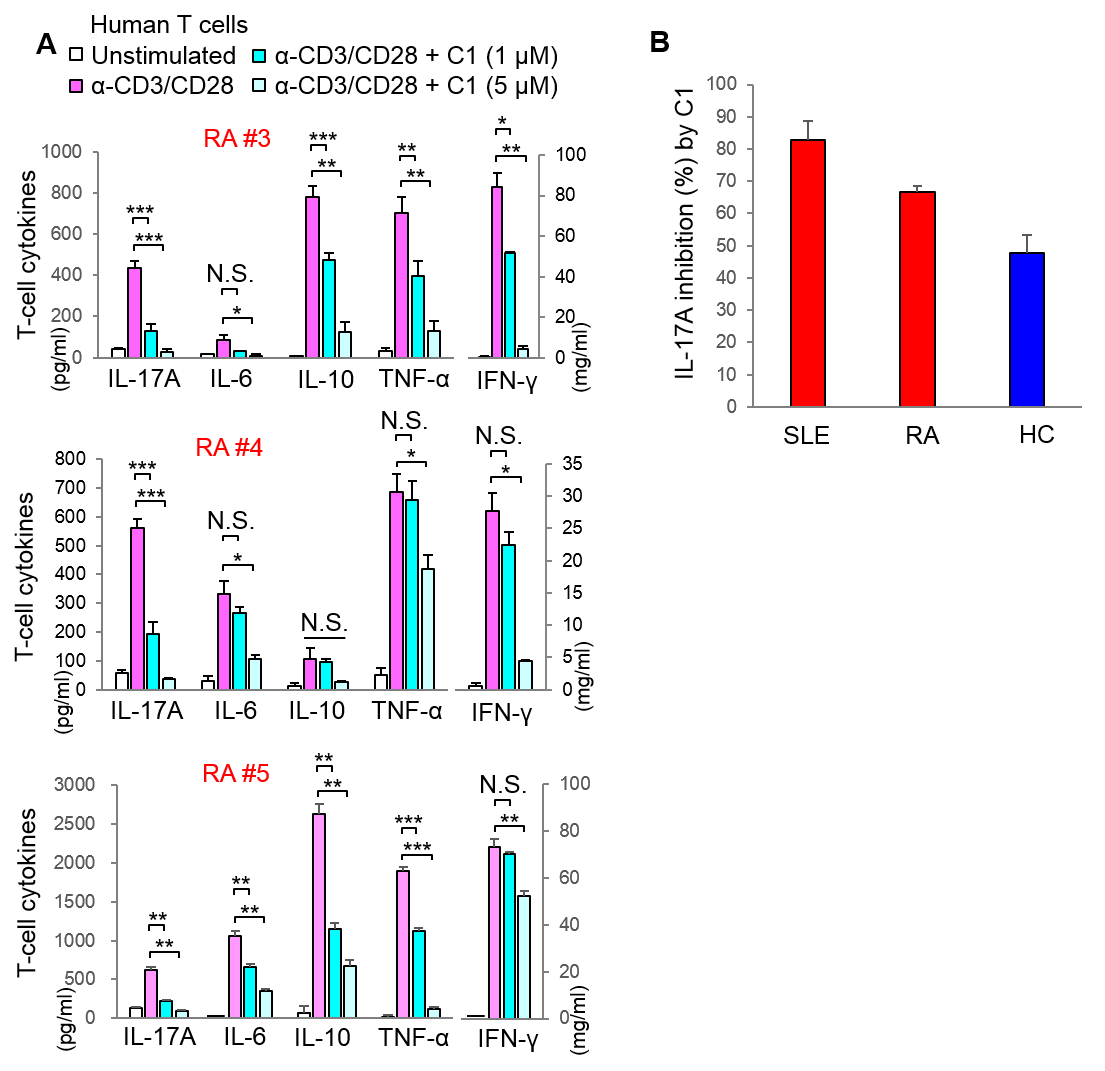


**Supplementary Figure 6. Inhibition of IL-17A overproduction by verteporfin in inflammatory T cells from SLE or RA patients.** (**A**) ELISA of various cytokines in supernatants of T cells from RA patients. T cells were stimulated with anti-CD3/CD28 and co-treated with verteporfin (C1) (1 or 5 μM) for 3 days. Means ± SD are shown. (**B**) The inhibition (%) of IL-17A by the 1 μM verteporfin (C1) treatment in SLE or RA patients was compared to that of healthy controls (HC). Means ± SEM of are shown. HC, healthy controls; SLE, systemic lupus erythematosus; RA, rheumatoid arthritis.
